# 10-MDP Based Dental Adhesives: Adhesive Interface Characterization and Adhesive Stability—A Systematic Review

**DOI:** 10.3390/ma12050790

**Published:** 2019-03-07

**Authors:** Eunice Carrilho, Miguel Cardoso, Manuel Marques Ferreira, Carlos Miguel Marto, Anabela Paula, Ana Sofia Coelho

**Affiliations:** 1 Faculty of Medicine, University of Coimbra, 3000-354 Coimbra, Portugal; eunicecarrilho@gmail.com (E.C.); mmferreira@fmed.uc.pt (M.M.F.); cmiguel.marto@uc.pt (C.M.M.); anabelabppaula@sapo.pt (A.P.); anasofiacoelho@gmail.com (A.S.C.); 2 Institute of Integrated Clinical Practice, Faculty of Medicine, University of Coimbra, 3000-354 Coimbra, Portugal; 3 Institute for Clinical and Biomedical Research (iCBR) area of Environment Genetics and Oncobiology (CIMAGO), Faculty of Medicine, University of Coimbra, 3000-354 Coimbra, Portugal; 4 CNC.IBILI, Faculty of Medicine, University of Coimbra, 3000-354 Coimbra, Portugal; 5 Institute of Endodontics, Faculty of Medicine, University of Coimbra, 3000-354 Coimbra, Portugal; 6 Institute of Experimental Pathology, Faculty of Medicine, University of Coimbra, 3000-354 Coimbra, Portugal

**Keywords:** 10-MDP, methacryloyloxydecyl dihydrogen phosphate, dental adhesives, self-etch adhesives, universal adhesives

## Abstract

The incorporation of functional monomers in dental adhesive systems promotes chemical interaction with dental substrates, resulting in higher adhesion forces when compared to micromechanical adhesion only. The 10-MDP monomer, whose chemical structure allows for a polar behavior which is favorable to adhesion, also promotes the protection of collagen fibers through the formation of MDP-calcium salts. This systematic review aimed to characterize the interface created by 10-MDP containing adhesive systems through an evaluation of the following parameters: Formation of nano-layered structures, capacity to produce an acid-base resistant zone, and adhesion stability. The research was conducted using PubMed, Cochrane Library, Web of Science and Embase, limited to English, Spanish, and Portuguese articles. The research was done according to the PICO strategy. The 10-MDP monomer has the capacity to produce an acid-base resistant zone on the adhesive interface, which increases the response to acid-base challenges. The adhesion established by these systems is stable over time. To have the best of these adhesive solutions, a scrubbing technique must be used to apply the adhesive system on dental substrates, in order to improve monomers infiltration and to create a stable bond. Time must be given for the solution to infiltrate, hybridize and form the MDP-Ca, improving adhesive stability.

## 1. Introduction

The procedures for performing a resin composite restoration include enamel etching, dentin conditioning, dentin priming and application of a dentin bonding agent, prior to the resin composite filling. However, since the introduction of adhesive resin-based restoration procedures, dental adhesives have been remarkably improved, and most commercially available adhesive systems have been simplified by combining some of the required steps [1,2,3]. In this context, and concerning direct dental restorations, self-etch adhesives and universal adhesives (systems which provide the operator with the choice for selecting the adhesive strategy; etch-and-rinse, self-etch or selective enamel etching) provide the promise for a specific chemical interaction capable of achieving more stable and long-lasting adhesion, with no additional tooth preparation for macro-mechanical retention, when compared to etch-and-rinse adhesives (whose principal adhesion mechanism is related to the micromechanical retention preceded by the removal of the smear layer) [4,5,6]. This interaction occurs due to functional monomers, which are acidic molecules that may serve various functions, such as etching tooth substrates (partially dissolving the smear layer and demineralizing hydroxyapatite), enhancing monomer penetration, and imparting the adhesives with potential for chemical interactions with dental substrates [7,8,9,10,11].

Functional monomers have at least one polymerizable group and a functional group which wets and demineralizes the substrate. According to the ‘adhesion-decalcification’ concept (the functional monomer either decalcifies or bonds to the tooth substrate), the functional group first ionically interacts with calcium in hydroxyapatite; depending on the resulting stability of the calcium-monomer complex, this ionic bond may either decompose and demineralize the tooth surface or remain stable [9,10,11,12].

Functional monomers have already been ranked based on their chemical bonding potential and 10-MDP (10-methacryloyloxydecyl dihydrogen phosphate) has been identified as being capable of establishing a very intensive and stable chemical interaction with hydroxyapatite. The MDP-Ca water-insoluble salts contribute to the protection of the collagen fibers. The atomic relation of the 10-MDP molecule favors the chemical interaction. However, impurities and dimers may reduce adhesive forces when using adhesive systems with this functional monomer [4,9,11,13,14,15].

The intense chemical interaction established between MDP and hydroxyapatite is ascribed to the superficial dissolution of hydroxyapatite induced by the adsorption of MDP, and subsequent deposition of MDP-Ca salts with lower solubility than of the salts produced by other functional monomers [14]. Monomer selection criteria must include properties such as calcium salt stability, wetting of the substrate and copolymerization behavior [16].

The aim for this article was to carry out a systematic review of the literature in order to evaluate the differences between the adhesive interfaces produced by dental adhesives containing 10-MDP and other adhesive systems. The aim was also to evaluate both bond strengths and adhesive stability of dental adhesives containing 10-MDP, in comparison with other systems/functional monomers.

## 2. Materials and Methods 

The protocol used for this systematic review followed the recommendations of Preferred Reporting Items for Systematic Reviews and Meta-Analyses Protocols (PRISMA-P) [17].

The research strategy of the present work was formulated according to PICO (Problem, Intervention, Comparison, Outcome) as seen in Table 1.

### 2.1. Search Strategy

A literature search was conducted in the Pubmed, Cochrane Library, Web of Science and Embase databases, using the search formulas described in Table 2. Only articles in English, Spanish or Portuguese, published until January 2019 were included.

### 2.2. Inclusion and Exclusion Criteria

The inclusion and exclusion criteria for selection and extraction of data are described in Table 3.

According to the predetermined inclusion and exclusion criteria, all titles and abstracts were examined by one reviewer (M.C.) in order to find relevant studies; the full texts of the relevant studies were scrutinized by two reviewers (M.C. and E.C.) independently to select eligible studies on the outcomes described in the PICO strategy. Any disagreement was discussed, and the opinion of a third reviewer (A.S.C.) was sought if necessary.

Studies on commercially available adhesive systems were included in order to understand the 10-MDP performance compared to other functional monomers. Studies on specific formulations, where several groups were formed, varying on the concentrations of their components, were also included so that the mechanism of each one could be described or highlighted.

For each proposed outcome and included study, descriptive and quantitative information was extracted, including authors, year of publication, control and test groups, results (quantitative and qualitative) and relevant conclusions.

Due to the disparity of methodology, it was not possible to perform a quantitative analysis (meta-analysis).

## 3. Results

The initial search resulted in 1383 references: 274 from PubMed, 9 from Cochrane Library, 711 from Web of Science and 389 from Embase.

**Figure 1:**
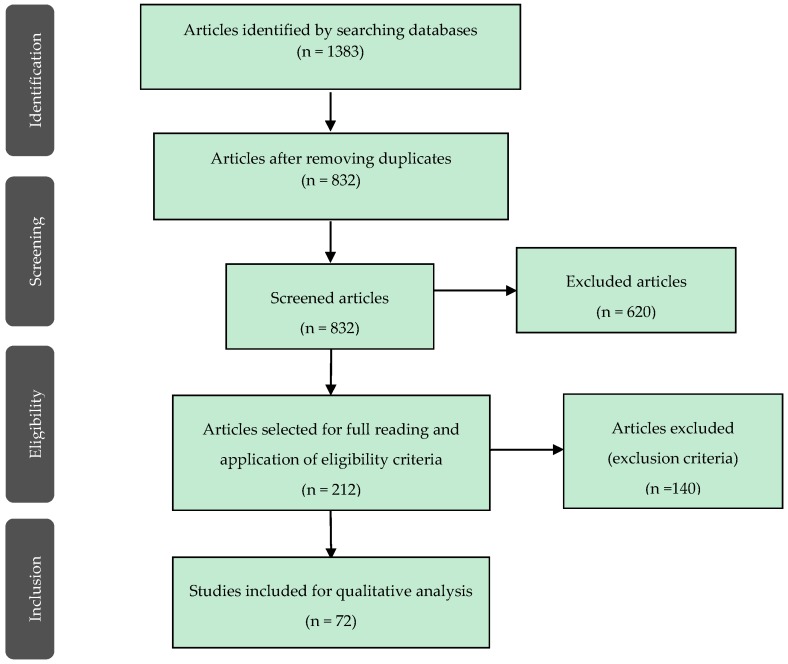
Search work-flow diagram.

After evaluating titles and abstracts, 212 relevant studies were obtained. After full-text analysis, 72 references were included in this systematic review (Figure 1).

The key characteristics evaluated were:

- Formation of nano-layered structures (MDP-Ca salts)—Formation/absence of nano-layered structures and morphology (Table 4);
- Acid-base resistant zone (ABRZ)—Formation or absence of ABRZ, thickness, and differences between dentin ABRZ and enamel ABRZ (Table 5);
- Adhesive stability—Measurement of adhesion forces (Table 6).

### 3.1. 10-MDP Monomer: Molecular Structure, Hydrophilicity and Nano-Layered Structures

Regarding the molecular structure of the 10-MDP monomer, its hydrophilicity and the formation of nano-layered structures, seven articles were found [5,6,18,19,20,21,22]. Commercially available adhesives systems [6,18,19,20] and experimental adhesive formulations [5,6,18,20,21,22] were used in order to clarify the different behavior between 10-MDP and other monomers used, as well as the different capacities regarding formation of nano-layering at the adhesive interface. The results are summarized in Table 4.

### 3.2. Capacity to Create an Acid-Base Resistant Zone (ABRZ)

Regarding the capacity of adhesive systems to produce an acid-base resistant zone six articles were found [2,10,23,24,25,26]. Commercially available adhesive systems [10,23,24,25,26] and experimental primer and bond [2,10,11,12,13,14,15,16,17,18,19,20,21,22,23] were used to clarify the capacity of 10-MDP to create an ABRZ compared to other commonly used functional monomers. The results are summarized in Table 5.

### 3.3. Adhesive Stability

Regarding 10-MDP-related adhesive stability 22 articles were found [1,8,10,11,12,13,15,23,25,27,28,29,30,31,32,33,34,35,36,37,38,39]. Commercially available adhesive systems [1,8,10,23,25,28,29,30,32,33,34,35,36,37,38,39] and experimental adhesive formulations [8,10,11,12,13,15,23,27,31,36,38] were used to evaluate the adhesive stability of the 10-MDP monomer. The results are summarized in Table 6.

## 4. Discussion

Self-etch and universal adhesive systems were introduced in dentistry to reduce and facilitate the clinical application of these biomaterials, by overcoming some etch-and-rinse disadvantages such as a greater number of steps, longer application time, technique sensitivity and difficulty in controlling dentin wetness [40]. However, these adhesion strategies work less favorably with enamel, as acid etching is not necessary in order to demineralize collagen fibrils. In etch-and-rinse adhesives that step might lead to several micrometers depth of demineralized substrate, especially in dentin, which is not completely hybridized by the bond solution of those systems, promoting degradation, a process initiated by nanoleakage [41]. In mild and ultra-mild self-etch adhesive systems, the abundant presence of hydroxyapatite remaining around the collagen fibrils provides natural protection to the collagen and allows the functional monomers to potentially interact with the substrate. Typical resin tags will only be formed when using strong self-etching adhesives. The potential interaction of self-etch adhesives depends on the surface-preparation method [41,42,43]. 

Functional monomers are not the only components in adhesive systems formulations so the clinical protocol for self-etching adhesives application cannot be the same for all the commercial systems: different solvents may require changes in the protocols (time, application, …) for better results. Active application of adhesives using a scrubbing technique promotes solvent evaporation, leading to the impregnation of a higher rate of monomers inside the smear layer, thus improving adhesive-interface quality. Solvent evaporation is also dependent on substrate characteristics (orientation of dentin surfaces) and on the uniformity of the adhesive layers. Long-term retention is achieved with high-quality chemical interaction between the adhesive and the substrate, through the formation of a hybrid layer, characterized as a three-dimensional collagen-resin biopolymer that provides a continuous and stable link between the adhesive and the dentin substrate; micromechanical retention may be additionally present when pre-etching the enamel or when using strong self-etching adhesive systems [27,34,43,44]. When talking about adhesive systems, interaction with collagen is probably the most important aspect, since the deterioration of collagen fibrils within the hybrid layer compromises the long-term stability of dentin bonding; the chemical properties of functional monomers are thought to account for the high bond strength with dentin [45].

Self-assembled nano-layered structures have been identified through adhesive interfaces of commercial self-etch and universal adhesive systems, both on enamel and dentin. These structures, which are typical of the 10-MDP monomer, are thought to produce a better water-stable interface which is favorable to adhesion, and may justify the higher adhesive stability of 10-MDP containing adhesive systems, along with the stable MDP-Ca salts [18,19,20,46]. Although nano-layered structures (which can be identified when 10-MDP based adhesives are used) are thought to play an important role in the adhesive stability bond strength, some doubts remain on the actual role of these structures. In fact, nano-layering cannot be responsible for durability of resin-dentin bond since it was not identified through all of the adhesive interfaces of the commercially available adhesive systems. These structures contribute to a higher resistance to biodegradation and to the longevity of the bond by enhancing the immediate performance of the adhesive systems [6,19,47,48].

Functional monomers give adhesive systems formulations the capacity to interact with dental substrates. However, functional monomers may decrease the degree of conversion of camphoroquinone/amine-curing adhesives; this decrease is monomer-dependent, meaning that a different degree of conversion was observed depending on the incorporated monomer and concentration used, but was reduced by simultaneous interaction of the functional monomer with hydroxyapatite [23,49,50]. Also, functional monomers are partially responsible for the hydrophobic/hydrophilic behavior of bonding resins [23,28,30]. Though more hydrophilic spacer carbon chain induces more water sorption and better dentin wettability, more hydrophobic functional monomers (MDP) are more suitable in order to avoid the effects of hydrolytic degradation [21,49,51].

Nanoleakage corresponds to defects at the resin-dentin interface from hydrolytic degradation, which may serve as pathways for degradation; double application self-etch adhesives may contribute to the durability of the bond by building a less permeable layer [33]. Also, applying the adhesive by employing a scrubbing technique enhances resin monomer infiltration of dentin, water chasing on the dentin surface and smear layer dissolution, improving the quality of the adhesive interface, especially on mild self-etching adhesive systems [34].

The 10-MDP monomer has a proven potential to interact with hydroxyapatite; the bond produced by 10-MDP containing adhesives appears to be very stable, as confirmed by the low dissolution rate of its calcium salts in water. Etching capacities are related to the substrate where it is applied, to the incorporated monomer and to the bonding potential of other commonly used functional monomers (4-META, phenyl-P). At different degrees the bonding potential is substantially low, or produces bonds which are not hydrolytically stable [52]. However, adhesion differentials between commercial adhesive systems are noticed depending both on the dental substrate and on other components included in the adhesives formulations. Some universal adhesives were found to produce poor adhesive interfaces by being less 10-MDP concentrated which suggests that an optimal concentration and purity of 10-MDP in self-etch and universal adhesives may exist so the maximum potential of this functional monomer is achieved [5,8,11,12,53]. 

The 10-MDP monomer has a long and hydrophobic spacer chain and creates a rich MDP-Ca salt adhesive interface, which improves adhesion strength, remaining stable after one year of water-storage [13,15,31,45,54]. Although all the advantages of this monomer, application protocols are crucial (substrate, time and technique) [34,55,56]. The application of an extra hydrophobic layer when using one-step self-etching or universal adhesive systems may improve the adhesive interface (in terms of durability and of resistance to degradation) and increase the long-term retention of restorative materials [57]. When using one bottle, self-etching or universal adhesive systems enamel etching may be recommended since these adhesive systems tend to have higher pH values, which lowers the ability to etch the enamel [58,59].

However, MDP-Ca salts were found to depend on the components that constitute commercial adhesives more strongly than on the concentrations of MDP and water in the adhesive [60]. Water concentration in adhesive systems was found to affect the efficacy of smear layer removal, and dentin bonding performance more strongly than the pH value of the adhesives [61,62] and ethanol was found to limit the dissociation of phosphate groups from the 10-MDP monomer [8]. 4-META was found to enhance both enamel and dentin bond-strengths more effectively than HEMA [63]. Although HEMA tends to improve bond strength, HEMA-free adhesives are preferred because of its hydrophilicity; on the other hand, HEMA brings solvents back into solution [1,54,64]; also, MDP-HEMA aggregates were found to compromise the MDP-collagen interaction leaving collagen fibrils unprotected by MDP and HEMA [21,65,66,67]. Other components may compete for calcium against 10-MDP, such as zinc ions [68]. Calcium hydroxide was found to improve the degree of conversion without interfering with bond strength to dentin, or the extent of nanoleakage [69].

Adhesive systems containing 10-MDP have a proven interest. However, it is important not to forget, when using strong self-etching adhesive systems, that the adhesive solution may penetrate into dentinal tubules and reach the pulp, especially when restoring deep cavities. Current studies on cytotoxicity lack a complete understanding of the effect of these materials on the pulp, because it is difficult to mimic the clinical conditions of its application. Some studies have reported that minimally toxic concentrations of 10-MDP promoted an inflammatory response and suppressed odontoblastic differentiation of dental pulp cells [70,71]. Also, chemical properties of MDP-containing adhesives alter during storage because MDP hydrolysis leads to acidification of the adhesive solutions [72,73].

## 5. Conclusions

When selecting a functional monomer or adhesive system, 10-MDP monomer appears to be a safe choice because of its molecular structure which is favorable to adhesion, its hydrophobic behavior and characteristic adhesive interface which favors bond durability and strength.

10-MDP containing dental adhesives are biomaterials which can establish strong and durable adhesive interfaces. Although 10-MDP has a proven capacity to interact with hydroxyapatite, some clinical steps of application of these adhesives are crucial for the resultant bond interface. 

To have the best of these adhesive solutions, selective enamel etching and a scrubbing technique must be used to apply the adhesive system on dental substrates, in order to improve monomers infiltration and to create a stable bond. Time must be given for the solution to infiltrate, hybridize and form the MDP-Ca, protecting collagen fibrils and improving adhesive stability.

## Figures and Tables

**Table 1:** PICO strategy.

P (Problem)	Permanent teeth with need for restoration.
I (Intervention)	Direct restoration with composite, using adhesives with 10-MDP.
C (Comparison)	Adhesives with different functional monomers other than 10-MDP. Different adhesives with 10-MDP monomer.
O (Outcome)	Capacity to create an acid-base resistant zone (ABRZ). Formation of nano-layered structures. Adhesive stability.

**Table 2:** Research strategy used.

Database	Search Strategy
PubMed	(“methacryloyloxydecyl dihydrogen phosphate” OR “10-MDP” OR “Functional monomer*”) AND (“dental cements [Mesh]” OR “adhesive*” OR “bond*”).
Cochrane Library	(“methacryloyloxydecyl dihydrogen phosphate” OR “10-MDP” OR “functional monomer*”) AND (“adhesive*” OR “bond*”).
Web of Science	TS = (“methacryloyloxydecyl dihydrogen phosphate” OR “10-MDP” OR “FUNCTIONAL MONOMER*”) AND TS = (“adhesive*” OR “bond*”).
Embase	(‘methacryloyloxydecyl dihydrogen phosphate’:ti,ab,kw OR ‘10-mdp’:ti,ab,kw OR’functional monomer*’:ti,ab,kw) AND (‘adhesive*’:ti,ab,kw OR ‘bond*’:ti,ab,kw).

**Table 3:** Inclusion and exclusion criteria.

**Inclusion Criteria**	Studies on permanent teeth
	Direct restorations
	Dental adhesives
**Exclusion Criteria**	Studies on deciduous teeth
	Indirect restorations
	Dental cements
	Adhesion to metal alloys, ceramics, posts
	Plaque inhibitors/Antibacterial activity
	Deproteinized dentin

**Table 4:** Formation of nano-layered structures (MDP-Ca salts).

Author, Year	Groups	Results
Yoshihara et al., 2011 [18]	T1*: mixed solution containing 15% 10-MDP C*: Clearfil SE Bond primer (Kuraray)	Nano-layering was stronger on dentin than on enamel; Rubbing the primer for 20 s enhanced nano-layering; Nano-layering was reduced with lower [MDP]
Yoshida et al., 2012 [19]	T1*: Clearfil SE Bond (Kuraray) T2*: Scotchbond Universal (3M ESPE)	Hybrid layer: T1* thicker than T2*; T1*—at the top of the hybrid layer, regular longitudinally layered structures, often curved; T2*—nano-layering near the tubule orifices where the adhesive infiltrated residual smear.
Yoshihara et al., 2013 [20]	T1: 2-MEP T2: 6-MHP T3*: 10-MDP T4: Adper Easy Bond (3M ESPE) T5*: All-Bond Universal (Bisco) T6*: Clearfil S3 Bond (Kuraray) T7*: Scotchbond Universal (3M ESPE)	T1—Results not obtainable (failure at preparation); T2—hybrid layer thinner than T3 and HAp-rich; T3*—thicker hybrid layer, intense nano-layering through the whole adhesive layer; Nano-layering formation for all 10-MDP based adhesives; T4 (6-MHP) only formed some nano-layering; In contrast to T4, the 3 commercially available 10-MDP containing adhesives revealed the 3 characteristic nano-layering peaks (XRD) already after 20 s interaction.
Hiraishi et al., 2014 [21]	T1*: 10-MDP T2: 4-META	10-MDP long chain makes it quite hydrophobic; Atelocollagen and MDP tend to aggregate in water; Reduction in the STD intensity when HEMA was added to T1*, resulting in a weak interaction with atelocollagen.
Yokota et al., 2015 [22]	T1*: Experimental adhesive	Several types of MDP-Ca salts and amorphous DCPD were developed during decalcification; Enamel and dentin produced MCS-MM and MD; Dentin produced DCS-MD.
Tian et al., 2016 [6]	T1*: 5% 10-MDP primer T2*: 10% 10-MDP primer T3*: 15% 10-MDP primer T4*: Adhese Universal (Ivoclar-Vivadent) T5*: All-Bond Universal (Bisco) T6*: Clearfil S3 Bond Plus (Kuraray) T7*: Clearfil SE Bond 2 (Kuraray) T8*: Clearfil Universal Bond (Kuraray) T9*: G-Premio Bond (GC Corp.) T10*: Scotchbond Universal (3M ESPE)	Nano-layering became sparser with reduction in [MDP] (T3* > T1*, T2*); Nano-layering was identified in limited sites when using T6* and T7*; T4*, T5*, T8*, T9*, T10*: no nano-layered structures were identified; T7*: limited and less well-organized patterns of nano-layering when compared to pure 10-MDP.
Yaguchi, 2017 [5]	T1*: 25.6 mg T2*: 49.9 mg T3*: 80.5 mg T4*: 116.1 mg (quantity of 10-MDP in 1g of the experimental adhesive)	↑ [10-MDP] on enamel led to ↑ production of MCS-MD and ↓ production of MCS-MM, and then it leveled; Dentin produced ↑ [MCS-MD] and [DCS-MD] than enamel did (*p* < 0.05); ↑ [10-MDP] on dentin led to ↑ production ratios of both MCS-MM and MCS-MD; Dentin showed a greater production of MDP-Ca salts than enamel did (*p* < 0.05); Dentin produced greater amounts of mono- and di-calcium salts of the MDP dimer that were able to form nano-layered structures; dentin and enamel predominantly produced a mono-calcium salt.

C: control group; DCS: di-calcium salt; DCPD: dicalcium phosphate dihydrate; HAp: hydroxyapatite; MCS: mono-calcium salt; MD: MDP dimer; MM: MDP monomer; STD: saturation transfer difference; T: test group; TEM: transmission electron microscopy; XRD: X-ray Diffraction; *: 10-MDP containing adhesive system/experimental adhesive.

**Table 5:** Acid-Base Resistant Zone (ABRZ).

Author, Year	Groups	Results
Na li et al., 2010 [10]	C*: 10-MDP in primer and bond (Clearfil SE Bond, Kuraray) T1*: 10-MDP (primer) and Phenyl-P (bond) T2*: Phenyl-P (primer) and 10-MDP (bond) T3: Phenyl-P in primer and bond	Enamel ABRZ thickness (µm): C*: 0.5; T1*: <0.2; T2*: 1; T3: <0.1. T1* ABRZ morphology similar to C* but sparser distribution of crystals; T2* crystals with ↑ length, but similar width (ABRZ), with clear intercrystallite spaces. T3 had low capacity to create an ABRZ and presence of funnel-shaped erosions; Adhesive interface produced by 10-MDP containing adhesive systems remained after acid-base challenge.
Nikaido et al., 2011 [2]	T1*: 10-MDP T2: 3D-SR T3: 4-META (similar compositions, different functional monomers, all universal adhesives)	Dentin ABRZ thickness: T1* > T2 > T3; Enamel ABRZ is very thin, compared to dentin ABRZ; Enamel ABRZ thickness < 0.5 µm in all groups but for T1* it appeared to be thicker. Dentin ABRZ formed under the hybrid layer, while enamel ABRZ was created along the interface between adhesive and enamel; ABRZ was confirmed at both enamel and dentin; it was influenced by the functional monomer contained in the adhesive system; Funnel-shaped erosion found at bonding interface between enamel and outer lesion in T3.
Nurrohman et al., 2012 [24]	C: Scothbond multi-purpose (3M ESPE) T2*: Clearfil photo bond (Kuraray) T3*: Clearfil SE Bond (Kuraray) T4: Adper Easy Bond (3M ESPE)	C: 4 µm HL and some regions with absence of a crystalline phase; deep funnel-shaped lesion into intact dentin; similar lesion in T2*; T2*: 5 µm HL and regions with low density and partially dissolved apatite crystals; T3*: 1 µm HL and denser overall crystallite arrangement in the base of the HL; approximately 0.5 µm thick ABRZ with densely arranged crystals and no funnel-shaped lesions in all specimens of this group; T4: partially demineralize HL, approximately 0.5 µm; funnel-shaped lesions along the apatite-rich zone.
Matsui et al., 2015 [23]	C*: Clearfil SE Bond (Kuraray) T1*: Experimental adhesive (10-MDP in primer)	Dentin ABRZ formed beneath the HL in both groups; Funnel-shaped erosion observed at the junction of dentin and bonding layer in T1*; Excluding 10-MDP from the bonding resin resulted in ↓ resistance against acid attack at ABRZ.
Nikaido et al., 2015 [26]	T1*: Clearfil SE Bond (Kuraray) T2*: Clearfil Bond SE One (Kuraray) T3: G-Bond Plus (GC)	T3 ABRZ was the thinnest (*p* < 0.05), and had the highest NL (*p* < 0.05); Funnel-shaped lesion not observed for T1*.
Guan et al., 2016 [25]	T1*: Clearfil SE Bond 2 (Kuraray) T2: Optibond XTR (KERR) T3*: Scotchbond Universal (3M ESPE), applied as SE, ERM (Moist) and ERD (Dry)	ABRZ at the front of demineralization for SE groups; Slope at bottom of outer lesion in T2; T3*SE: funnel-shaped lesion at bottom of outer lesion; T3*ERM and T3*ERD: 5 µm HL without appearance of ABRZ.

ABRZ: acid-base resistant zone; C: control group; ER: etch-and-rinse; HL: hybrid layer; NL: nanoleakage; SE: self-etch; T: test group; *: 10-MDP containing adhesive system/experimental adhesive.

**Table 6:** Adhesive stability.

Author, Year	Groups	Results	Comments
Hayakawa et al., 1998 [27]	T1: 5% Phenyl-P + 60% H_2_O T2: 10% Phenyl-P + 55% H_2_O T3: 20% Phenyl-P + 45% H_2_O T4: 30% Phenyl-P + 35% H_2_O T5*: 5% 10-MDP + 60% H_2_O T6*: 10% 10-MDP + 55% H_2_O T7*: 20% 10-MDP + 45% H_2_O T8*: 30% 10-MDP + 35% H_2_O	Dentin T3, T4—30 s treatment: ↑TBS than T1 and T2 (*p* < 0.05); Dentin T8*—15 s treatment: ↑adhesion than T1, T2, T3 (*p* < 0.05); Dentin T8*—60 s treatment: ↑adhesion than T5*; Different patterns after treatment with T1/T4 and T5*/T8*.	Adhesives partially dissolved the smear layer which restricted the resin penetration. Monomers could infiltrate into the dentin to create the hybrid layer, resulting in a tight adhesion to dentin; Insufficient infiltration of monomers into the dentin, preserving more of the smear layer, resulted in lower BS.
Inoue et al., 2005 [28]	T1*: Clearfil SE Bond (Kuraray) T2: Unifil Bond (GC) T3: Clearfil Liner Bond II (Kuraray)	T1*: µTBS to dentin after 100,000 thermocycles = 0 thermocycles; T2: ↓µTBS (41%) after 100,000 thermocycles; T3: ↓µTBS (48%) after 30,000 and 100,000 thermocycles; HAp crystals remained at the hybrid layer (T1* > T2 > T3).	Long-term durability of the dentin-adhesive interface of two-step self-etching adhesives differed, depending on the particular adhesive; T1* showed no signs of degradation in bond strength and interfacial ultrastructure.
Na Li et al., 2010 [10]	C*: Clearfil SE Bond (Kuraray) T1*: 10-MDP (primer) and Phenyl-P (bond) T2*: Phenyl-P (primer) and 10-MDP (bond) T3: Phenyl-P in primer and bond	C*: ↑BS than the other groups (*p* < 0.005); T1*, T2*, T3: no differences in BS (*p* > 0.05); Significant distribution of failure modes among groups (*p* < 0.05); C*, T1*, T2*: adhesive and cohesive failure, while major failure of T3 was adhesive failure; Micro-shear bond-strength values showed ↑bond strength in C (*p* < 0.005). Among test groups, no significant difference was found.	
Fujita et al., 2011 [29]	T1*: Clearfil Tri-S Bond (Kuraray) T2*: Clearfil SE Bond (Kuraray)	T2*: ↑ [reacted 10-MDP] (16.1%) compared to T1 (9.2%); T1*: blank outline of the enamel prisms; dentinal tubes were widened, with deposits on the intertubular dentin, without exposure of collagen fibrils; T2*: typical etching pattern on enamel; dentinal tubes were more widened and blocked by precipitates, with collagen fibrils exposed; conditioning of enamel and dentin allowed enhancement in the initial BS (*p* < 0.05); a reduction was observed in conditioned dentin after 20,000 thermocycles.	Superior BS of T2* correlated to the demineralized amount of tooth apatite by 10-MDP; Unreacted 10-MDP polymer within the adhesive layer did not ↓ the bond strength, despite application of 20,000 thermocycles.
Harnirattisai et al., 2012 [30]	T1*: Clearfil SE Bond (Kuraray) T2*: Clearfil Tri-S Bond (Kuraray) T3: G-Bond (GC) T4: i-Bond (Kulzer) (T2, T3, T4: all-in-one adhesives)	Bond strength at 10 min was lower than that at 24 h for all adhesives; T1*: ↑bond strength (10 min and 24 h); SBt: ↑adhesive failure (66.04–97.44%) for all-in-one adhesives, compared to T1* (10 min and 24 h); µSBt: ↑cohesive failures in resin; µSBt: T1* > T4 > T2* and T1 = T3.	Dentin cohesive failure was found to be lower in the µSBt of T1 at 24 h; µSBt results in divergency of behavior between systems, not seen with SBt;
Iwai et al., 2012 [12]	T1*: 0 mg T2*: 25.6 mg T3*: 49.9 mg T4*: 80.5 mg T5*: 116.1 mg (quantity of 10-MDP in 1g of experimental adhesive)	↑ [10-MDP] resulted in ↑amounts of MDP-Ca salts, which resulted in ↑BS for enamel and dentin; Further ↑ in the amount of MDP-Ca salt resulted in ↓BS.	
Zhang et al., 2013 [8]	C: Durafill Bond (Heraeus Kulzer) T1*: MDP/HEMA/Bis-GMA (1:1:1) (Kuraray) T2*: MDP/HEMA/Bis-GMA (2:1:1) (Kuraray) T3*: MDP/Bis-GMA (1:1) (Kuraray)	µTBS: C lower than test groups (24 h and 1 year water-storage) (*p* < 0.05); No differences between test groups (*p* > 0.05); C: clearly visible enamel HAp crystallites partly and adhesive mixed with fractured HAp crystallites partly (solely micromechanical interlocking at the interface); Test groups: Faintly visible enamel HAp crystallites partly.	Etched enamel surfaces treated with the MDP-containing primers revealed that the etched enamel surfaces were covered by a layer of variable network-like/fibril-like HAp crystallites; C: cannot chemically react with HAp
Feitosa et al., 2014 [13]	T1: MEP T2*: MDP T3: MDDP T4: CAP-P T5: MTEP	T2*, T3: lowest free-calcium concentrations (*p* < 0.001); T1 had the highest; Monomer-Ca salt on dentin present in all groups; T2*, T3: ↑µTBS than T1, T4, T5 (*p* < 0.05).	Formation of monomer-Ca salts and initial BS were influenced by the length and hydrophilicity of the spacer chain of functional monomers.
Feitosa et al., 2014 [15]	T1: MEP T2*: MDP T3: MDDP T4: CAP-P T5: MTEP	T1: lowest monomer-calcium formation (*p* < 0.05); T2*, T3: ↑µTBS than those of T1, T4, T5; After 1-year aging: drop in µTBS was observed for T5 (enamel and dentin), T1 (enamel) and T4 (enamel) (*p* < 0.005); T5: highest micro-permeability; T1, T4, T5: ↑NL after aging.	Length and hydrophilicity of the spacer chain influenced the monomer-calcium salt formation, the dentin/enamel bonding performance, the interfacial micro-permeability and NL.
Takahashi, 2014 [31]	T1*: 0 g; T2*: 3.0 g; T3*: 6.0 g; T4*: 10.0 g; T5*: 15.0 g. (quantity of 10-MDP in 1 g of the experimental adhesive)	T1*: thermocycling led to a ↓ in the BS, with no MDP-Ca salt produced (*p* < 0.05); ↑ of MDP-Ca salts to above: - 37.2 mg/g: ↓ dentin BS during thermocycling; - 57.9 mg/g: difference in dentin BS before and after thermocycling (*p* < 0.05); - 57.9 mg/g: dentin exhibited more changes in the surface morphology than enamel and in the type of fracture mode during thermocycling; ↑ of MDP-Ca salts changed the morphology of the fractured enamel surface and ↑ the number of specimens that had less than half of the adhesive remaining at the enamel or dentin surface.	
Anchieta et al., 2015 [32]	C: Scotchbond Multi-Purpose (3M ESPE) T1*: Clearfil SE Bond (Kuraray) T2: One Up Bond F (Tokuyama) T3: Adper Easy One (3M ESPE) T4: Filtek LS adhesive (3M ESPE)	C: thickest hybrid layer (*p* < 0.05); longest resin tags ( = T2) (*p* < 0.05); T1*: thinnest hybrid layer; highest elastic modulus of the hybrid layer (*p* < 0.05); T2: thinnest adhesive layer (*p* < 0.05); highest degree of silver impregnation at 24 h (*p* < 0.05); T3, T4: highest infiltration (*p* < 0.05). T4: thickest adhesive layer; Storage for 12 months ↑ silver impregnation for all groups (*p* < 0.05), except for T1* (*p* > 0.05); ↓Elastic modulus along time in all groups (*p* < 0.05); ↑ NL over time except for T1*.	Partially demineralized dentin below the hybrid layer occurred for all adhesives; After 12 months storage, degradation occurred at the DAI in all groups and the intensity of degradation differed depending on the type of adhesive used; 10-MDP containing adhesive system (T1*) DAI formed showed the best stability among all adhesive systems.
Matsui et al., 2015 [23]	T1*: Clearfil SE Bond (Kuraray) T2*: Experimental adhesive with 10-MDP primer	T1* µTBS > T2* µTBS without thermocycling (*p* < 0.001); after thermocycling: T2* > T1* (*p* < 0.001); T2* µTBS remained stable after thermocycling (*p* < 0.001); UTS: T2* > T1* in all evaluation periods; ↓ UTS after storage in water.	
Muñoz et al., 2015 [33]	C1: Adper Single Bond 2 (3M ESPE) C2*: Clearfil SE Bond (Kuraray) T1: Peak Universal Adhesive System (Ultradent Products Inc.) T2*: Scotchbond Universal Adhesive (3M ESPE) T3*: All Bond Universal (Bisco) C1, T1, T2 and T3 as ER C2, T1, T2 and T3 as SE	Most of the specimens showed adhesive or adhesive/mixed failures; T1 (SE) and T1 (ER) showed the ↑ immediate µTBS, similar to C1, C2* (*p* > 0.05) with a ↓ after 6-months of water storage (*p* > 0.05); T2* (SE), T2* (ER), T3* (SE), T3* (ER): lower immediate µTBS, compared to C1, C2* (*p* < 0.05); ER: only T3* had lower µTBS after 6-months (*p* < 0.05); T1: highest NL at immediate time (*p* < 0.05), ↑ after 6 months (*p* < 0.05);	Universal adhesives demonstrated heterogenous behavior, since some adhesives diminished the bonding performance over the course of time.
Yoshihara et al., 2015 [11]	Three 10-MDP molecules by different companies: T1*: 83% purity T2*: 90% purity T3*: ↑% than T1 and T2	T1*: µTBS did not ↓ after 100,000 thermocycles, contrarily to T1* and T2*; T3*: ↑Immediate µTBS than T1*, T2*; No pre-testing failure recorded for T3*, but several failures happened with the “aged specimens” of T1* and T2*.	Differences in the ultrastructure of the hybrid layer were observed between the different monomers used.
Chen et al., 2015 [36]	T1: Prime and Bond Elect (Dentsply) T2*: Scotchbond Universal (3M ESPE) T3*: All Bond Universal (Bisco) T4*: Clearfil Universal Bond (Kuraray) T5: Futurabond U (VOCO)	Comparisons between test groups were all significant (*p* < 0.01), except between T1/T2*, T1/T3*, T2*/T3*, T4*/T5; T2* (*p* = 0.004), T4* (0.006) and T5 (*p* < 0.001) had different results between with and without thermocycling; T1 and T3* were resistant to thermocycling (*p* > 0.01).	
Farias et al., 2016 [37]	T1*: Scotchbond Universal (3M ESPE) T2*: All Bond Universal (Bisco) T3: Optibond FL (Kerr) T4: Adper Single Bond Plus (3M ESPE) T5*: Clearfil SE Bond (Kuraray) T6: Adper Prompt L-Pop (3M ESPE)	Similar µTBS means, before and after thermocycling for T1*, T2*, T3, T4 (*p* < 005); Before thermocycling: similar µTBS means between groups: T1*/T2*ER/T3/T4/T5* (*p* < 0.05), T1*SE/T2*SE/T3/T6(*p* < 0.05) T1*SE/T2*SE/T3/T5* (*p* < 0.05). After thermocycling: similar µTBS means between groups T1*/T2*ER/T3/T4/T5* and T1*SE/T2*SE/T3/T4.	
Tsuchiya et al., 2016 [38]	T1*: Clearfil SE Bond (Kuraray) T2: Experimental adhesive (equal to T1*, without MDP)	T1*: ↑SB (*p* < 0.05) with pre-etching, for same storage period; ↑SB at 6-months and 1-year storage; ↑SFS (*p* < 0.05) with pre-etching for same storage period; ↑SB for both test groups at 6 months storage.	
Zhang et al., 2016 [1]	T1*: All-Bond Universal (Bisco) T2*: Clearfil Universal Bond (Kuraray) T3: Futurabond U (VOCO) T4: Prime&Bond Elect (Dentsply) T5*: Scotchbond Universal (3M ESPE)	µTBS was affected by the bonding strategy and aging tests (*p* < 0.005); 12 months: ↑µTBS for T1* as ER (*p* < 0.001), while for the rest of the groups was ↑ when in SE mode (*p* < 0.001); T1* to T5*: ↓µTBS when in ER mode (*p* < 0.001); T1*, T2*, T3: ↓µTBS when in SE mode (*p* < 0.001); T4, T5*: no changes in µTBS between 24 h and 12 months.	Universal adhesive systems with 10-MDP monomer did not show better performance than those without; Bonds created in SE mode were more durable than those created in ER mode; With exception of bonds created by T4 and T5, universal adhesives at test were incapable of defying ageing.
Thanatvarakorn et al., 2016 [34]	T1*: Clearfil SE Bond (Kuraray) T2*: Scotchbond Universal (3M ESPE) T1*s, T2*s: applied with scrubbing technique T1*ns, T2*ns: passively applied	T1*s did not affect µTBS (*p* > 0.05); T2*s exhibited ↑µTBS than T2*ns (*p* < 0.05); T2*ns: adhesive failure at adhesive interface was predominant and larger than in other groups; T2*s had the highest etching ability, while T2*ns had the lowest; T1*s and T2*s were free of NL.	Scrubbing technique not only improved immediate µTBS but also ↑ the stability of a one-step self-etching adhesive bond to dentin.
Guan et al., 2016 [25]	T1*: Clearfil SE Bond 2 (Kuraray) T2: Optibond XTR (KERR) T3*: Scotchbond Universal (3M ESPE), applied as SE, ERM (Moist) and ERD (Dry)	↓ 24 h BS of ER than SE groups (*p* < 0.05); 5000 thermal cycles: ↓µTBS of T3*ERM (*p* = 0.001); 10,000 thermal cycles: T1* µTBS remained stable, T2 ↑ and all T3* ↓ (*p* < 0.05); Immediate BS of T3*ERD was lower than other groups (*p* < 0.05); 10,000 thermal cycles: ↓BS on SE and ER (*p* < 0.05);	
Tsujimoto et al., 2017 [39]	T1*: Clearfil Universal Bond (Kuraray) T2*: G-Premio Bond (GC) T3*: Scotchbond Universal (3M ESPE) T4*: Clearfil SE Bond (Kuraray) T5*: Clearfil SE Bond 2 (Kuraray) T6: Optibond XTR (Kerr)	Initial BS: T6 > T4* > T5* > T3* > T1* > T2*; SFS: T6 > T4* > T5* > T3* > T1* > T2*; Initial BS of universal adhesives is influenced by the type of adhesive, but lower than that of two-step self-etching adhesive systems.	
Wang et al., 2017 [35]	T1*: Clearfil SE Bond (Kuraray) T2*: Scotchbond Universal (3M ESPE) T3: Optibond XTR (Kerr) T4: Adper Easy Bond (3M ESPE)	TF-XRD: T1* and T2* revealed production of 10-MDP-Ca salts; T2*: slightly shifted and ↓intensity; no detected peaks in T3 and T4; SEM: T1*, T2*: after ethanol rinsing most of the adhesive was retained; T4: smear debris remained; T3: all of the hybrid layer was removed; T1* and T2* µTBS stable before and after thermocycling (*p* > 0.05); Dentin µTBS: T2* and T3 ↑ than T1* and T4 at 24 h; T4 ↓ after thermocycling and T3 after aging; NL: T1 and T2—slight ↑ impregnation after thermocycling; T3 and T4: ↑ infiltration after thermocycling and in many cases the entire length of the hybrid layer was infiltrated.	Differences in T1* and T2* for TF-XRD analysis are related to the ratio of 10-MDP contained in each formulation; T2* and T3 gained ↑ bonding strength even after aging than the traditional T1* and T4, although T3 showed ↑ NL after thermocycling.

BS: Bond strength; C: Control group; DAI: Dentin-adhesive interface; HAp: Hydroxyapatite; NL: Nanoleakage; NMR: NMR spectroscopy; SB: Shear bond; SBt: Shear bond test; SFS: Shear fatigue strength; T: Rest group; TBS: Tensile bond strength; TF-XRD: Thin-film X-ray diffraction; UTS: Ultimate tensile strength; µSB: Micro-shear bond; µSBt: Micro-shear bond test; µTBS: Micro-tensile bond strength; *: 10-MDP containing adhesive system/experimental adhesive.
